# Anti-SARS-CoV-2 Immunoglobulin Isotypes, and Neutralization Activity Against Viral Variants, According to BNT162b2-Vaccination and Infection History

**DOI:** 10.3389/fimmu.2021.793191

**Published:** 2021-12-17

**Authors:** Maciej Tarkowski, Wilco de Jager, Marco Schiuma, Alice Covizzi, Alessia Lai, Arianna Gabrieli, Mario Corbellino, Annalisa Bergna, Carla Della Ventura, Massimo Galli, Agostino Riva, Spinello Antinori

**Affiliations:** ^1^ Department of Biomedical and Clinical Sciences L. Sacco, Università degli Studi di Milano, Milan, Italy; ^2^ Luminex B.V., Hertogenbosch, Netherlands; ^3^ Division of Infectious Diseases, Aziende Socio Sanitarie Territoriali (ASST) Fatebenefratelli Sacco Hospital, Milan, Italy

**Keywords:** SARS-CoV-2, COVID-19, coronavirus, multiplex immunoassay, bead, fluorescence, serological assay, neutralization assay

## Abstract

**Purpose:**

To compare SARS-CoV-2 antigen-specific antibody production and plasma neutralizing capacity against B.1 wild-type-like strain, and Gamma/P.1 and Delta/B.1.617.2 variants-of-concern, in subjects with different Covid-19 disease and vaccination histories.

**Methods:**

Adult subjects were: 1) Unvaccinated/hospitalized for Covid-19; 2) Covid-19-recovered followed by one BNT162b2 vaccine dose; and 3) Covid-19-naïve/2-dose BNT162b2 vaccinated. Multiplex Luminex^®^ immunoassays measured IgG, IgA, and IgM plasma levels against SARS-CoV-2 receptor-binding domain (RBD), spike-1 (S), and nucleocapsid proteins. Neutralizing activity was determined in Vero E6 cytopathic assays.

**Results:**

Maximum anti-RBD IgG levels were similar in Covid^-^19‑recovered individuals 8‒10 days after single-dose vaccination and in Covid-19-naïve subjects 7 days after 2^nd^ vaccine dosing; both groups had ≈2‑fold higher anti-RBD IgG levels than Unvaccinated/Covid-19 subjects tracked through 2 weeks post-symptom onset. Anti-S IgG expression patterns were similar to RBD within each group, but with lower signal strengths. Viral antigen-specific IgA and IgM levels were more variable than IgG patterns. Anti-nucleocapsid immunoglobulins were not detected in Covid-19-naïve subjects. Neutralizing activity against the B.1 strain, and Gamma/P.1 and Delta/B.1.617.2 variants, was highest in Covid‑19-recovered/single-dose vaccinated subjects; although neutralization against the Delta variant in this group was only 26% compared to B.1 neutralization, absolute anti-Delta titers suggested maintained protection. Neutralizing titers against the Gamma and Delta variants were 33‒77% and 26‒67%, respectively, versus neutralization against the B.1 strain (100%) in the three groups.

**Conclusion:**

These findings support SARS-CoV-2 mRNA vaccine usefulness regardless of Covid-19 history, and confirm remarkable protection provided by a single vaccine dose in people who have recovered from Covid-19.

## Introduction

On March 11, 2020, the World Health Organization (WHO) declared the novel coronavirus disease 2019 (Covid-19) outbreak a global pandemic (1). As of October 11, 2021, the Covid-19 pandemic has caused almost 238 million infections worldwide, resulting in 4.9 million deaths (2, 3). Coronaviruses are enveloped single-stranded RNA viruses that share structural similarities and are composed of 16 non-structural proteins and four structural proteins: the homotrimeric spike (S), envelope, membrane, and nucleocapsid (N) proteins (4–6). Common to other coronaviruses, severe acute respiratory syndrome coronavirus 2 (SARS-CoV-2) entry into host cells is mediated by S glycoproteins projecting from the viral surface. The S protein’s S1 subunit contains the receptor-binding domain (RBD) sequence that specifically binds angiotensin-converting enzyme 2 (ACE-2) on host cells to allow virus entry (4–10).

Infection with SARS-CoV-2 generates antibody responses even in critically ill patients and despite having low circulating lymphocyte counts (11). Both IgG and IgA responses differ in their levels and durability between mild and severe Covid-19 patients, whereby higher levels of IgG and IgA against viral S1 protein occurred in people with severe Covid-19 (12, 13). Quantitative Ig changes correlate with titers of neutralizing antibodies (nAbs) primarily in patients exhibiting severe Covid-19 symptoms but not in moderate or mild disease (14). Both anti-S-IgG antibodies and neutralization activity are detected in sera of SARS-CoV-2-infected people 5‒7 days after symptom onset, rapidly increase and peak around Day 40, and begin declining thereafter (14). Neutralizing activity may vary in a 2-phase pattern, because hospitalized individuals showed an initial rapid decay until Day 80 post-symptom onset, and a slower decrease afterwards, with detectable activity still at 6 months (15). Deceased patients mounted a robust yet delayed production of anti-S, and anti-RBD IgG and nAb levels compared to survivors, and this delay correlated with impaired viral control (16). A firm understanding of the seroconversion kinetics after SARS-CoV-2 infection is essential to managing the Covid-19 pandemic.

The BNT162b2 vaccine (Pfizer–BioNTech) is a nucleoside-modified messenger RNA that encodes the S protein of SARS-CoV-2 (17). In adults, a series of two 30-µg intramuscular injections of BNT162b2 spaced 3 weeks apart resulted in high titers of nAbs against SARS-CoV-2, and strong T-cell-mediated responses specific to the viral S protein (18, 19). An ongoing global randomized trial of volunteers ≥16-years-old demonstrated 95% effectiveness in preventing Covid-19 beyond 7 days after receiving the second vaccine dose, with an acceptable safety profile (20). These results supported the December 11, 2020 US Food and Drug Administration (FDA) emergency use authorization (EUA) for the BNT162b2 vaccine in individuals aged ≥16-years (21). In a younger cohort of 12‒15-year-olds, BNT162b2 vaccination demonstrated a similar safety profile with 100% effectiveness in preventing Covid-19 (22), and was the basis for the May 10, 2021 EUA expansion to include individuals aged ≥12 years (23).

Reports published soon after global Covid-19 vaccination began confirmed the vaccine efficacy observed in clinical trials. It also became clear that people previously exposed to the SARS-CoV-2 virus responded to a single vaccine dose by producing very high levels of virus-specific antibodies that were not additionally increased after the second dose (24, 25). In previously infected patients, antibody levels against SARS-CoV-2 after one dose were significantly higher than in naive patients fully vaccinated 3‒4 weeks after second dose (26). These results led to the decision in Italy, France, and Germany to administer a single dose of vaccine to some people previously exposed to SARS-CoV-2. Antibody-mediated protective responses to infection may depend on Covid-19 symptom severity (27), and even asymptomatic or mildly symptomatic individuals might respond strongly to single-dose vaccination (28). A deeper comprehension of variations in Covid-19 vaccine protective responses in persons with prior Covid-19 of differing severity will be important for selecting the best approach for Covid-19 prophylaxis in those individuals.

It remains uncertain how long Covid-19 vaccination protection endures in different populations, and this knowledge becomes even more important with the emergence of new SARS-CoV-2 variants (29). Particular concern exists with the Beta variant (B.1351, South African origin) and the Delta variant (B.1.617.2, Indian origin), against which Covid-19 vaccination confers a measure of protection although reduced compared to previously detected SARS-CoV-2 strains such as the Alpha variant (B.1.1.7, UK origin) (30, 31).

The current observational study used a multiplex bead-based system to simultaneously measure and compare plasma levels of antibodies directed against the SARS-CoV-2 S, RBD, and N regions in plasma from adult Italian subjects who were: 1) SARS-CoV-2-infected but never vaccinated; 2) SARS-CoV-2-infected with BNT162b2 vaccination initiated after recovery; and 3) Covid-naïve but BNT162b2 vaccinated. Isotypes IgG, IgM, and IgA were separately evaluated. We also performed neutralization assays with these same plasma samples to evaluate and compare their inhibition of SARS-CoV-2 infectivity of the B.1 strain, and Gamma and Delta variants-of-concern.

## Methods and Materials

### Subjects and Samples

All subject data were anonymized as required by the Italian Data Protection Code (Legislative Decree 196/2003) and the general authorizations issued by the Italian Data Protection Authority. Ethical oversight of this study was provided by the IRB of the University of Milan’s Luigi Sacco Hospital. All subjects provided written informed consent. This study conformed to the tenets of the Declaration of Helsinki.

Subjects comprised 19 adults who either: 1) were diagnosed with Covid-19 but did not undergo vaccination; 2) recovered from Covid-19 and received 1 dose of BNT162b2; or 3) had no history of SARS-CoV-2 infection and underwent the usual 2-dose regimen with BNT162b2.

The unvaccinated Group 1 were hospitalized patients at the Sacco University Hospital, Department of the Infectious Diseases, who had SARS-CoV-2 infection confirmed by RT-PCR. Time 0 for these subjects was defined as the date of symptom onset. The infected/recovered Group 2 were volunteer healthcare workers at our hospital, and received a single vaccination at least 3 months after Covid-19 symptom resolution and with a negative RT-PCR test. Time 0 for these volunteers was defined as date of vaccination. The Covid-19-naive/2-vaccine dose Group 3 also comprised volunteer healthcare workers at our institution, and study Time 0 was defined as the date of the first vaccination dose. Per Italian law, no financial or other compensation was provided to study subjects. Venous blood samples were drawn into K_2_-EDTA anticoagulant tubes between March 2, 2020 and March 15, 2021 and plasma aliquots were stored at -20°C until use.

### Blood Sampling Timing

We determined blood sampling timing based on previously reported peak Ig levels in prior studies of subjects with varied SARS-CoV-2 infection and vaccination histories. We had the most latitude in the largest group of previously uninfected subjects who received two vaccine doses, and planned regular sampling dates up to the 21 day booster and out an additional 3 weeks, per the large BNT162b2 regulatory trials (18–22). We had less sampling flexibility with the SARS-CoV-2 infected patients. Because virus incubation time averages approximately 1 week before symptoms appear, we opted to test through 14 days post-symptom onset, *i.e.*, an estimated 21 days after infection. In Covid-19 affected/recovered individuals, a single vaccine dose generates a large antibody response much more rapidly than either natural infection or a second vaccination dose in SARS-CoV-2 naïve individuals, with maximum antibody levels reported within 7 to 14 days at latest (24–26). Thus, we selected 10 days post-vaccination as a timepoint likely to capture maximal antibody responses to a single vaccine dose in previously infected individuals.

### Luminex Multiplex Immunoassays

Antibody levels against S, RBD, and N proteins of Covid-19 were simultaneously measured in human plasma samples using the Luminex xMAP^®^ SARS−CoV−2 Multi-Antigen IgG kit (Product #30-00124; Luminex Corp., Austin, TX), a fluorescence bead-based multiplex assay that has received FDA authorization under an EUA (US FDA 2020; Available at: https://www.fda.gov/media/140257/download). Assay 96-well plates were read on a MAGPIX^®^ automated plate reader using xMAP MULTI IgG CoV-2 Assay Software (both from Luminex). For IgM and IgA antibody level determinations, the xMAP^®^ kit’s phycoerythritin (PE)-conjugated anti-IgG detection antibody was substituted with either goat anti-human IgM-PE (1:500) or goat anti-human IgA-PE (1:250) (respective products #GTIM-001 and GTIA-001; MOSS, Pasadena, MD); appropriate isotype-specific IgM and IgA control reagents are already included as standard Luminex IgG kit components.

### Cell Culture and Virus Propagation

SARS-CoV-2 isolation was performed as previously detailed (32). Briefly, Vero E6 cells derived from African green monkey kidney epithelium (ATCC; Accession #CRL-1586) were propagated in 75-cm^2^ tissue culture flasks using DMEM containing antibiotics and 10% fetal bovine serum (FBS), and monitored using an inverted light microscope. B.1 strain, and Gamma and Delta SARS-CoV-2 variants were isolated from patient swab samples and propagated in Vero E6 cells using DMEM + antibiotics with 2% of fetal bovine serum (FBS). All procedures using SARS-CoV-2 were performed under Biosafety Level 3 containment in accordance with institutional safety guidelines.

Cultures were observed after 24, 48, 72, and 96 hours for evidence of cytopathic effects. Culture supernatant was collected at each timepoint to perform RT-PCR. Viral RNA was extracted from 200 μL of culture supernatant with a QIAamp Viral RNA kit (Qiagen, Venlo, Netherlands), and eluted in 50 μL of water for use as the template for RT-PCR. For qPCR, Luna^®^ Universal One-Step RT-qPCR (New England BioLabs, Ipswich, MA) and SARS-CoV-2 (2019-nCoV) CDC qPCR Probe Assay (Integrated DNA Technologies, Coralville, IA) kits were used.

SARS-CoV-2 amplicons were obtained using two different primers pools (sequences at: https://artic.network/ncov-2019), purified with AMPure XP beads (Beckman Coulter, Indianapolis, IN) and checked for size using TapeStation 200 (Agilent Technologies, Santa Clara, CA). Amplicon libraries for Illumina deep sequencing was prepared using an Illumina DNA Prep and IDT ILMN DNA/RNA Index kit (Illumina, San Diego, CA. Library concentration was determined with the Invitrogen Quant-iT Picogreen dsDNA assay (Fisher Thermo Scientific, Waltham, MA). Resulting libraries were normalized and pooled for subsequent sequencing on an Illumina MiSeq platform using a 2×200 cycle paired-end sequencing protocol.

Results were mapped and aligned to the SARS-CoV-2 reference genome on the Global Initiative on Sharing Avian Influenza Data (GISAID) website (https://www.gisaid.org/, accession ID: EPI_ISL_406800). Consensus sequences were generated using Geneious software (v.9.1.5; Biomatters, Auckland, New Zealand). SARS-CoV-2 sequences were classified using the Pangolin COVID-19 Lineage Assigner tool v.2.3.2 (https://pangolin.cog-uk.io/) and Nextclade v.0.14.1 (https://clades.nextstrain.org/). SARS-CoV-2 full-length genomes were submitted to GISAID (Accession IDs: EPI_ISL_1085167, EPI_ISL_2472918, and EPI_ISL_2840619 for Alpha, Gamma, and Delta variants, respectively).

### Neutralization Assays

Vero E6 cells were trypsin-detached and cultured in flat-bottom 96-well plates at 5×10^3^ cells/200 µL media/well. At cell confluence, media were changed to DMEM containing 2% FBS, which was the culture media in subsequent neutralization assay steps. Preliminary virus titration experiments with serial dilutions of virus-containing supernatants determined variant-specific viral concentrations in cell supernatant that caused 50% Vero E6 cytopathy at 3 days, and were used for subsequent neutralization assays. Virus neutralization was performed in checkerboard fashion using serial dilutions of plasma to pre-treat serial dilutions of SARS-CoV-2 variants for 1 hour. Functional neutralization assays were then performed by transferring plasma-treated virus suspensions to flat-bottom 96-well plates containing confluent Vero E6 cells. Untreated virus (no plasma) was added to other Vero E6-containing wells as positive controls of viral infectivity and culture media-only served as negative controls (no cytopathy expected). All conditions were performed in duplicate wells. After 24 hours, media were exchanged with fresh media and cells were observed then and again at 48, 72, and 96 hours. The lowest concentration (highest dilution) of each human plasma sample that resulted in 100% protection of Vero E6 cells from lysis/cytopathy in the time that the virus dose alone caused 50% cell cytopathy was reported as that plasma sample’s neutralization titer against that particular SARS-CoV-2 viral strain.

### Data Analysis

Data were analyzed using Prism v.5 graphing and statistical analysis software (GraphPad Software, San Diego, CA), and Excel v.16 (Microsoft, Redmond, WA). Analyte concentrations are qualitatively presented as the average of median fluorescence intensity (MFI) units generated by the plate reader, mean neutralization titer ( ± SEM), or fold- or percentage difference, as appropriate. Means were compared by unpaired Student t-test, with two-tailed p-values <0.05 considered indicative of statistically significant differences.

## Results

### Subjects

We enrolled 8 subjects aged 20‒87 years who were hospitalized for Covid-19 infection (**Table 1**). We also recruited 12 volunteer healthcare workers aged 28-63 years from the same hospital unit, 3 with prior documented and resolved SARS-CoV-2 infection, and 9 persons who were not thought to have been previously infected. Of these 9 “Covid-19-naïve” individuals screened for inclusion, one subject, a 57-year-old male, displayed inordinately elevated anti-SARS-CoV-2 antibody background levels consistent with prior infection. Review of this person’s study documents revealed a history of recent exposure to a Covid-19-infected individual, and a subsequent RT-PCR test (non-diagnostic) indicated SARS-CoV-2 positivity; this person’s data were excluded from analysis, leaving n=8 volunteers in the previously uninfected/2 vaccination group.

**Table 1:** Subject Demographics.

Parameter	Infected, No Vaccine	Infected + Vaccine	Uninfected + Vaccine
N	8	3	8
Gender, male/female, n	6/2	1/2	3/5
Age, years, mean ± SD	61 ± 18	55 ± 1	40 ± 14
Age, years, range	43‒87	54‒55	28‒63

### Anti-SARS-CoV-2 Antibody Levels

**Figure 1:**
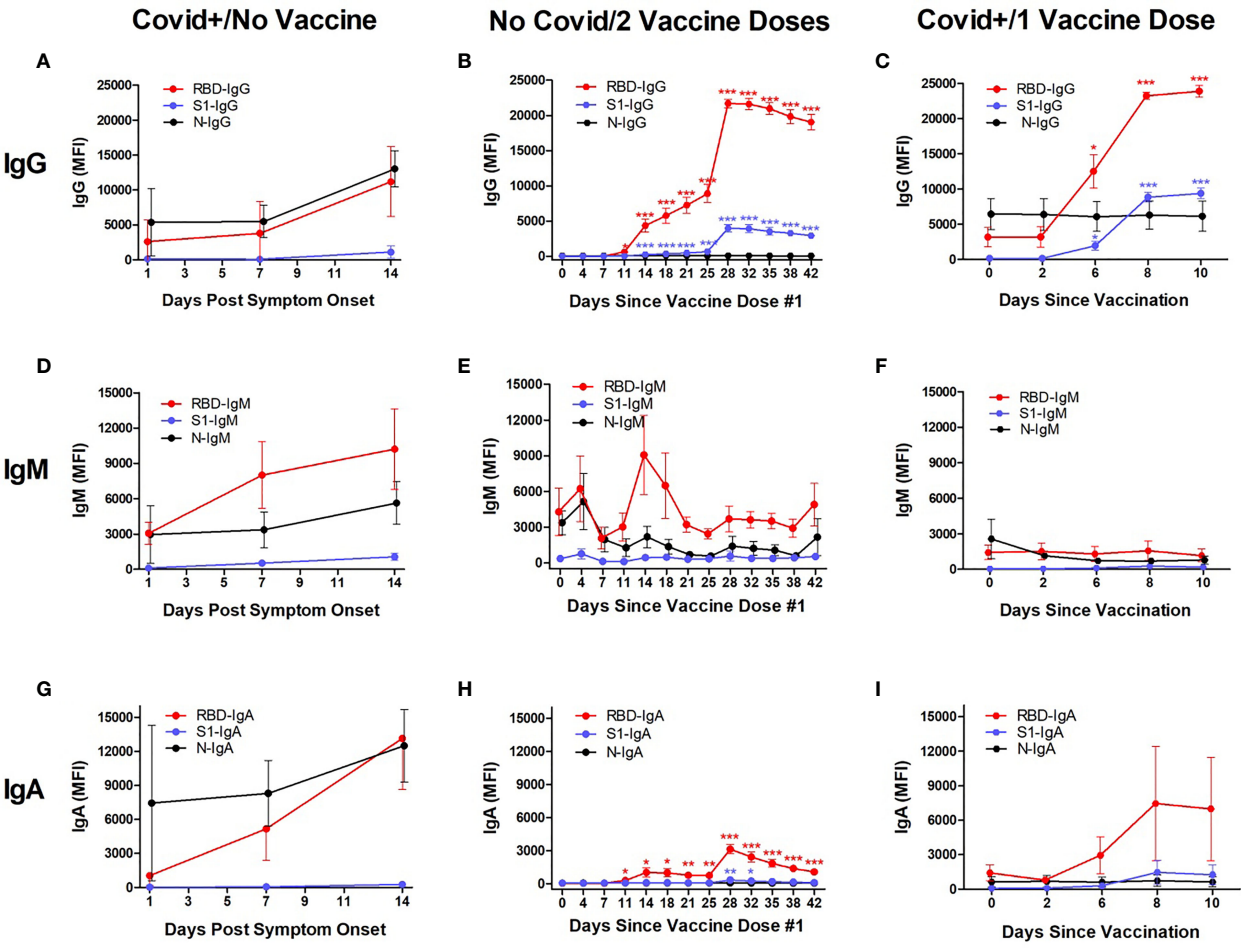
Kinetic measurement of plasma anti-SARS-CoV-2 IgG, IgM, and IgA isotype levels against Receptor Binding Domain (RBD), Spike Protein-1 Subunit (S1), and the Nuclocapsid (N) proteins in different populations. Shown are values in subjects who were hospitalized for Covid-19 infection, measured at timepoints after first reported symptom onset and before vaccination (“Covid+/No Vaccine”; n=8; left column), hospital workers who had no known SARS-CoV-2 exposure and were vaccinated with the Pfizer BNT162b2 vaccine, measured at timepoints after their first vaccination dose (“No Covid/2 Vaccine Doses”; n=3; middle column), and hospital workers who had recovered from Covid-19 and then received a single dose of BNT162b2, measured at timepoints after vaccination (“Covid+/1 Vaccine Dose”; n=8; right column). All individuals in the No Covid/2 Vaccine Dose group received their second vaccination 21 ± 1 days after the first dose. In Covid+/No Vaccine individuals, IgG, IgM and IgA levels were elevated against RBD and N at 2 weeks, whereas no S1-reactivity was observed **(A, D, G)**. The maximum anti-RBD IgG levels were seen at Day 14 after symptoms appeared (11,185 MFI). The No Covid/2 Vaccine individuals showed significantly but modestly elevated IgG **(B)** and transiently elevated IgM against RBD **(E)** at 2‒3 weeks after their first vaccination, with anti-S1 IgG upregulated by 14 days **(B)**, but no anti-S1 IgM or IgA during this time **(E, H)**. Starting ≈1 week after the 2nd vaccine dose, IgG against RBD rapidly increased to a maximal level at Day 28 (21,695 MFI), at which point it plateaued **(B)**; IgG against S1 also began increasing in parallel to a maximum level at 28 days (3964 MFI) followed by a plateau through Day 42 **(B)**. In the Covid+/1 Vaccine group, IgG against RBD and S1 began increasing at 6 days after vaccination and were maximal for both RBD (23,897 MFI) and S1 (9387 MFI) at 10 days **(C)**. No IgM response against viral RBD, S1, or N was observed in this group through 10 days after single-dose vaccination **(F)**. Significant IgA responses against RBD and S1 were not observed in the two groups that had been infected with SARS-CoV-2 **(G, I)**, but were seen in Covid-naive subjects after vaccination **(H)**. Antibody production against N was not seen in Covid-naïve groups **(B, E, H)**. “MFI” indicates average of median fluorescence intensity units. Shown are mean values ± SEM. Asterisk *p<0.05; **p<0.01; ***p<0.001 by unpaired t-test.

Anti-SARS-CoV-2 antibody levels (IgG, IgM, and IgA) were tracked for 14 days after reported symptom onset in 8 patients hospitalized for Covid-19 infection who had never received a SARS-CoV-2 vaccination, for 42 days after 1^st^ vaccination in 8 volunteers who had no SARS-CoV-2 exposure history and received both doses of the BNT162b2 series, and for 10 days after vaccination in 3 volunteers who had recovered from Covid-19 and then received a single BNT162b2 dose (**Figure 1**).

**IgG:** All three study groups displayed increased anti-RBD IgG levels after developing Covid-19 or receiving SARS-CoV-2 vaccinations, but antibodies against the S1 protein were only elevated in the two groups who received vaccine doses (**Figures 1A–C**).

In Covid+/No Vaccine subjects, plasma IgG levels against RBD and N did not significantly change through 1 week after Covid-19 symptom onset but both were elevated by 2 weeks, whereas no S1-reactivity was observed through the 2-week study period (**Figure 1A**). Maximum mean anti-RBD IgG levels were 3-fold increased between Day 7 (3803 ± 1596 MFI) and Day 14 (11,185 ± 2045 MFI) (p=0.014, n=8 and 6 for Days 7 and 14, respectively). The difference in mean anti-N IgG levels between Day 7 (5498 ± 2315 MFI; n=8) and Day 14 (13,031 ± 2559 MFI; n=6) approached but did not attain statistical significance (p=0.051).

Both groups that received vaccinations showed increased mean IgG levels against RBD and S1, but not N (**Figures 1B, C**). In the eight No Covid/2 Vaccine Doses subjects, anti-RBD IgG began steadily increasing 25-fold from baseline levels (25 ± 3 MFI) by Day 11 after 1^st^ vaccination (629 ± 215 MFI; p=0.014), and then rapidly increased between Days 25‒28 (booster vaccine administered on Day 21) to a maximum level 864-fold greater than starting levels (21,602 ± 815 MFI; p<0.001), with values remaining high but slowly decreasing through Day 42 (**Figure 1B**). Anti-S1 IgG increases were similar to anti-RBD IgG elevation in the No Covid/2 Vaccine group, with baseline anti-S1 IgG values (28 ± 6 MFI) significantly increasing by Day 14 (244 ± 30 MFI; p<0.001) and then rapidly increasing between Days 25‒28 to a maximum level (3964 ± 560 MFI; p<0.001), with values remaining elevated but slowly decreasing through Day 42. Anti-N IgG remained indistinguishable from background values at all timepoints in this group.

In the three Covid+/1 Vaccine subjects, IgG against both RBD and S1 increased quickly after a single SARS-CoV-2 vaccination (**Figure 1C**). Mean baseline anti-RBD IgG levels (3179 ± 1381 MFI) were significantly 4-fold elevated by Day 6 after vaccination (12,503 ± 2391 MFI; p=0.028), with maximal 7-fold increase reached by Day 8 (23,259 ± 527; p<0.001) that was maintained through Day 10 (23,897 ± 843 MFI; p<0.001). Mean anti-S1 IgG was increased 13-fold from baseline (155 ± 56 MFI) by Day 6 (1953 ± 585; p=0.038), 55-fold by Day 8 (8850 ± 693 MFI; p<0.001), and 61-fold elevated at Day 10 (9387 ± 762 MFI; p<0.001). Baseline anti-N IgG levels (6448 ± 2218 MFI) were not significantly changed through Day 10 (6132 ± 2139 MFI; p=0.923).

**IgM:** In Covid+/No Vaccine subjects, mean plasma IgM levels against RBD were numerically increased 3-fold from the day of symptom onset (3062 ± 954 MFI, n=2) through Day 14 (10,201 ± 3435 MFI; n=6) but this difference was not significant (p=0.300; **Figure 1D**). Similarly, baseline anti-N IgM values (2958 ± 2447 MFI) was 2-fold higher by Day 14 (5628 ± 1810 MFI) but not significant (p=0.475). Mean anti-S1 IgM levels at baseline (112 ± 46 MFI) were numerically increased 5-fold by Day 14 (1061 ± 306 MFI; p=0.141).

In the eight No Covid/2 Vaccine Doses subjects, baseline anti-RBD IgM levels (4297 ± 1986 MFI) trended towards a transient 2-fold increase at Day 14 after the first vaccine dose (9083 ± 3336 MFI), but this was not significant (p=0.238; **Figure 1E**). Variations in anti-S1 IgM and anti-N IgM were unremarkable through Day 42.

In the three Covid+/1 Vaccine subjects, no notable changes in IgM recognizing RBD, S1, or N were detectable through 10 days after vaccination (**Figure 1F**).

**IgA:** In Covid+/No Vaccine subjects (**Figure 1G**), mean plasma IgA levels against RBD were numerically increased 3-fold from the day of symptom onset (3062 ± 954 MFI, n=2) through Day 14 (13,158 ± 4508 MFI; n=6) but this difference was not significant (p=0.300). Similarly, baseline anti-N IgM values (2958 ± 2447 MFI) was 2-fold higher by Day 14 (5628 ± 1810 MFI) but not significant (p=0.475). Mean anti-S1 IgM levels at baseline (112 ± 46 MFI) were insignificantly increased 5-fold by Day 14 (1061 ± 306 MFI; p=0.141).

In the eight No Covid/2 Vaccine Doses subjects (**Figure 1H**), baseline anti-RBD IgA levels (46 ± 7 MFI) became significantly 6-fold elevated by 11 days after the 1^st^ vaccine dose (284 ± 91 MFI; p=0.021), which peaked at 68-fold above baseline on Day 28 (3128 ± 421 MFI; p<0.001), before gradually tapering to become 23-fold elevated (1059 ± 189 MFI; p<0.001) on Day 42. Anti-S1 IgA significantly but transiently increased and peaked at 5-fold over baseline (63 ± 45 MFI) beginning on Day 28 after the 1^st^ vaccination (336 ± 74 MFI; p=0.007), and had returned to baseline by Day 35 (183 ± 55 MFI; p=0.113). No IgA reactivity against N was detected.

In the three Covid+/1 Vaccine subjects (**Figure 1I**), anti-RBD IgA was numerically elevated from baseline (1424 ± 688 MFI) by a maximum of 5-fold at Day 8 after 1^st^ vaccination (7454 ± 4978 MFI), but this difference was not significant (p=0.296). Reactive IgA was not detected against S1 or N at any timepoint.

The No Covid/2 Vaccine individuals showed significantly but modestly elevated IgG (B) and transiently elevated IgM against RBD (E) at 2‒3 weeks after their first vaccination, with no evidence of S1 reactivity by any Ig isotype (B, E, H). Starting ≈1 week after the 2nd vaccine dose, IgG against RBD rapidly increased to a maximal level at Day 28 (21,695 MFI), at which point it plateaued (B); IgG against S1 also began increasing in parallel to a maximum level at 28 days (3964 MFI) followed by a plateau through Day 42 (B). In the Covid+/1 Vaccine group, IgG against RBD and S1 began increasing at 6 days after vaccination and were maximal for both RBD (23,897 MFI) and S1 (9387 MFI) at 10 days (C). An IgA response only occurred in the two groups that had been infected with SARS-CoV-2 (G, I), and was not seen in Covid-naïve subjects after vaccination (H). Antibody production against N was not seen in Covid-naïve groups (B, E, H).

### Maximal IgG Levels Against RBD and S1

**Figure 2:**
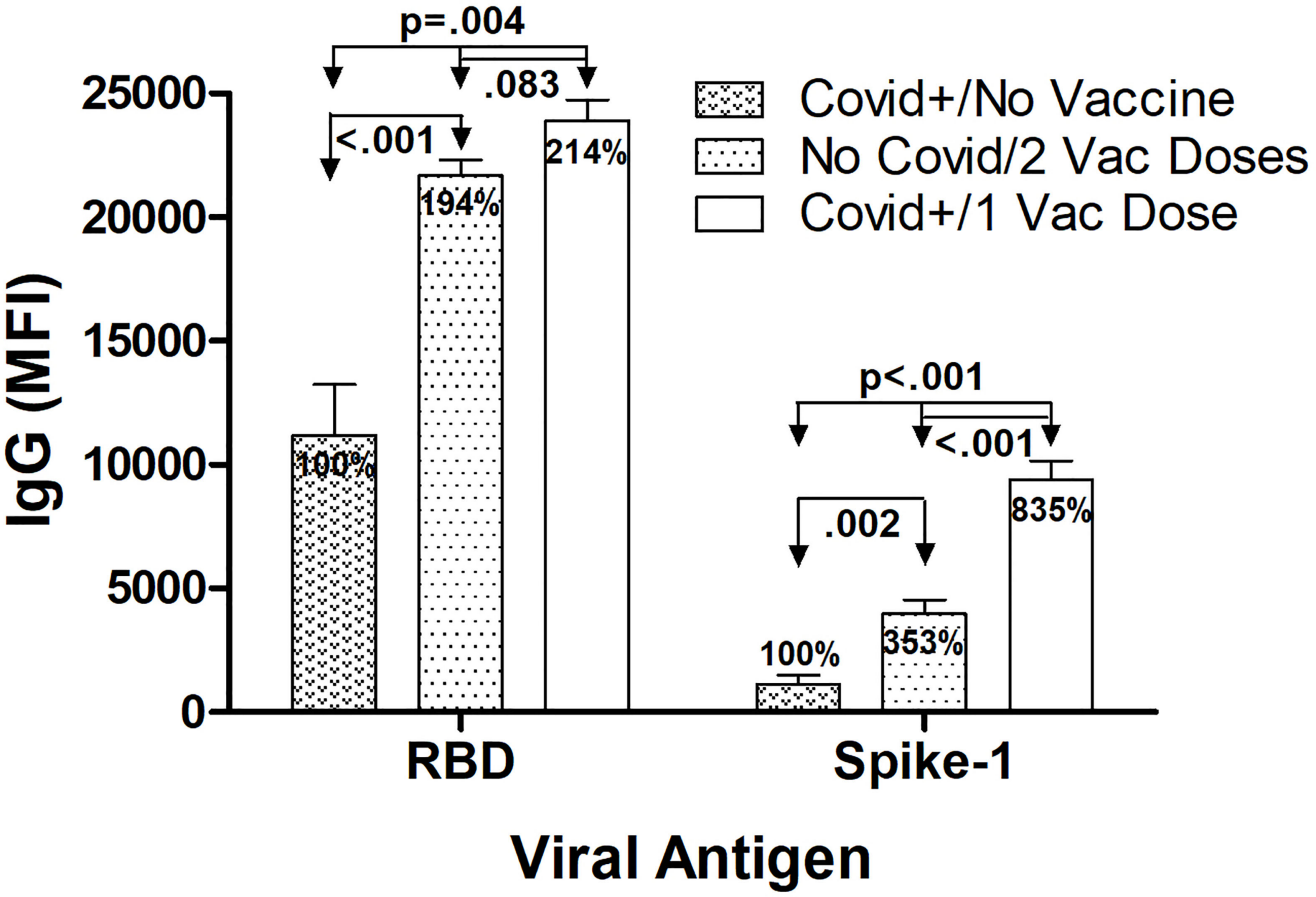
Highest IgG levels against RBD and S1 according to infection and BNT162b2 vaccination history. Maximum average anti-RBD IgG levels were highest 14 days after symptom onset in the SARS CoV-2 infected/unvaccinated subjects, 10 days after single vaccination in the Covid recovered/1 Vaccine Dose group, and 28 days after 1st vaccination (7 days after 2nd vaccine dose injection) in the No Covid/2 Vaccine Doses group. Anti-RBD IgG levels were significantly elevated in both vaccinated groups compared to in SARS-CoV-2 infected/unvaccinated individuals, with levels in both No Covid/2 Vaccine subjects and Covid+/1 Vaccine subjects elevated approximately 2-fold. A similar relative increase in anti-S1 IgG levels after vaccination also occurred, with levels in No Covid/2 Vaccine subjects over 3 fold higher and levels in Covid+/1 Vaccine subjects more than over 8-fold higher, respectively, compared to anti-S1 IgG measured in unvaccinated Covid-infected individuals. With S1 but not RBD, a single vaccination after previous Covid-19 infection resulted in significantly higher S1 specific IgG levels than occurred in Covid-naïve individuals who received the complete 2 dose vaccination series. Shown are mean values ± SEM for 3‒8 subjects/group.

The highest mean IgG levels measured against RBD and S1 occurred 14 days after symptom onset in the SARS-CoV-2 infected/unvaccinated subjects, 10 days after single vaccination in the Covid-recovered/1 Vaccine Dose group, and 28 days after 1^st^ vaccination (7 days after booster vaccination) in the No Covid/2 Vaccine Doses group (**Figure 2**). Anti-RBD IgG levels were significantly elevated in both vaccinated groups compared to SARS-CoV-2 infected/unvaccinated individuals, with levels 94% higher in No Covid/2 Vaccine subjects (p<0.001) and 114% elevated in Covid+/1 Vaccine subjects (p=0.004). This relative increase in IgG after vaccination was also observed for anti-S1 IgG, with levels 253% higher in No Covid/2 Vaccine subjects (p=0.002) and 735% higher in Covid+/1 Vaccine subjects (p<0.001), respectively, compared to anti-S1 IgG measured in unvaccinated SARS-CoV-2-infected individuals. The highest anti-S1 IgG levels observed in Covid-recovered individuals that received a single BNT162b2 dose were 2.4-fold higher than levels in Covid-naïve subjects who received the complete 2-dose vaccination series (p<0.001). While maximal anti-RBD IgG levels measured in the Covid+/1 Vaccine group were numerically 10% higher than anti-RBD IgG levels in the Covid-naïve/2 Vaccine group, this difference did not attain statistical significance (p=0.083). It is important to appreciate that in a multiplex immunoassay, differences in detection antibody specificity and binding kinetics do not allow direct quantitative comparison between, for example, measured RBD levels and S1 levels.

### SARS-CoV-2 Neutralization

**Figure 3:**
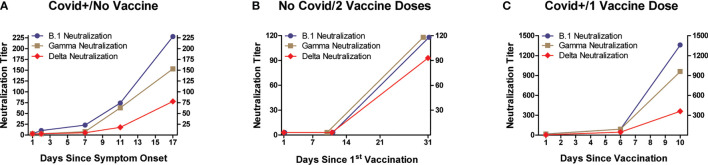
Neutralization of SARS-CoV-2 infectivity by plasma, according to subject infection and BNT162b2 vaccination history. Three viral variants (B.1 wild type-like early strain, Gamma, and Delta) were pre-incubated with serial dilutions of subject plasma before evaluating their infectivity of Vero E6 cells (ATCC #CRL-1586; African green monkey kidney epithelium). Y-axes denote plasma neutralization titers. All three experimental groups displayed variably increasing neutralization activity against the three SARS-CoV-2 variants, with timing of maximal observed neutralization approximating that when maximum anti-RBD and anti-S1 IgG levels were measured (detailed in **Figure 1**). The highest mean neutralization titers (and anti-RBD and anti-S1 IgG levels) were observed in individuals who received a single vaccine dose after recovering from Covid-19.

**Figure 4:**
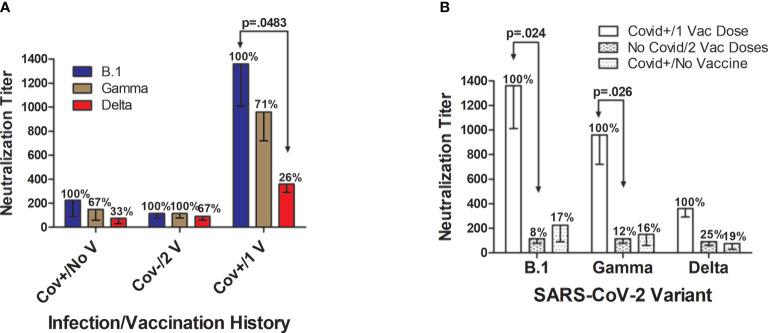
Neutralization of SARS-CoV-2 infectivity according to viral variant and subject infection and BNT162b2 vaccination history. **(A)** In unvaccinated individuals hospitalized with SARS-CoV-2 (n=8), mean neutralization effectiveness against the B.1 strain (set as 100%; titer=225 ± 135 fold dilution) was reduced by 33% against the Gamma variant (titer=150 ± 90) and by 67% against the Delta variant (titer=75 ± 45). In SARS-CoV-2-naïve subjects (n=8) who received the 2-dose BNT162b2 vaccination series, neutralizing effectiveness was similar against the three SARS-CoV-2 variants with inhibitory activity against the B.1 strain (set at 100%; titer=115 ± 39) maintained at 100% against the Gamma variant (titer=115 ± 39), and modestly 23% lower against the Delta variant (titer=90 ± 30). By far the strongest neutralizing ability was elicited in volunteers (n=3) who had previously been diagnosed with and recovered from Covid-19 and then received a single BNT162b2 vaccine dose. In this Covid+/1 Vaccine Dose group, the average maximal neutralizing titer against the B.1 strain (set at 100%; titer=1360 ± 349) were reduced by 29% against the Gamma variant (titer=960 ± 240; p>0.05) and by 74% against the Delta variant (titer=360 ± 69; p=0.048). **(B)** Individuals who had previously recovered from Covid-19 and then received a single vaccine dose developed significantly higher neutralization titers against B.1 and Gamma strains when compared to Covid-naïve individuals who received 2 vaccine doses. Neutralization of the Delta variant was also numerically 4-fold higher in Covid+/1 Vaccine Dose subjects versus Covid-naïve/2 Vaccine Dose subjects, and 5-fold higher than in unvaccinated individuals who were hospitalized for Covid-19, but these differences did not attain statistical significance. Neutralization capacity of the plasma of subjects hospitalized with Covid-19 did not remarkably differ from neutralization by Covid-naïve individuals who received the both vaccine doses, for all SARS-CoV-2 strains studied. Shown are mean values ± SEM.

Plasma samples were evaluated for neutralization effectiveness against infectivity by three SARS-CoV-2 variants (**Figures 3**, **4**). Three viral variants (B.1 wild type-like early strain, Gamma, and Delta) were pre-incubated with subject plasma before testing in an *in vitro* viral neutralization assay. Individuals infected with SARS-CoV-2 and hospitalized for Covid-19 developed increased neutralization titers against all three variants in concert with increased anti-RBD and anti-S1 IgG levels after symptom onset (**Figure 3A**). In SARS-CoV-2-naïve subjects (n=8) who received the 2-dose BNT162b2 vaccination series, neutralization also paralleled IgG level increases against RBD and S1 and highest observed values were recorded approximately 10 days after receiving the 2^nd^ vaccine dose, and were higher than levels in subjects relying on natural immunity alone (**Figure 3B**). The highest neutralization titers and anti-viral IgG levels were measured in individuals who had recovered from previous SARS-CoV-2 infection and subsequently received a single BNT162b2 dose (**Figure 3C**). In this last group, antibody levels were highest by 10 days after vaccination, and absolute values for both neutralization titers and anti-viral IgG levels were the highest of the three experimental groups.

Maximum plasma neutralizing titers against SARS-CoV-2 infectivity differed according to viral variant and subject infection and vaccination history (**Figure 4**). In unvaccinated individuals hospitalized with SARS-CoV-2, neutralization against the B.1 strain in comparison was reduced by 33% against the Gamma variant and by 67% against the Delta variant (**Figure 4A**). In SARS-CoV-2-naïve subjects who received two BNT162b2 vaccinations, neutralizing effectiveness was similar against the three SARS CoV-2 variants with inhibitory activity against the B.1 strain and Gamma variant being essentially identical, with comparative neutralization against the Delta variant slightly 23% lower. In the Covid+/1 Vaccine Dose group, the average maximal neutralizing titer against the B.1 strain was reduced by 29% against the Gamma variant (titer=960 ± 240; p>0.05) and by 74% against the Delta variant (titer=360 ± 69; p=0.048).

By far the strongest viral neutralizing ability was elicited in volunteers with a documented prior Covid-19 diagnosis who received a single BNT162b2 vaccination after recovery (**Figures 4A, B**). Covid+/1 Vaccine Dose subjects had significantly higher neutralization titers against B.1 (p=0.024) and Gamma strains (p=0.026) when compared to Covid-naïve individuals who received 2 vaccine doses (**Figure 4B**). Neutralization activity against the Delta variant was 4-fold greater in the Covid-recovered/1 Vaccine Dose subjects versus Covid-naïve/2 Vaccine Dose individuals, and 5-fold higher than in unvaccinated individuals hospitalized for Covid-19; however, these differences did not reach mathematical significance.

## Discussion

As we approach the 2^nd^ year of the SARS-CoV-2 pandemic (1), while numerous therapies remain under investigation for treating Covid-19 (33), vaccination remains the mainstay approach for reducing the rates of infection, severe disease, and mortality across the globe (34). Understanding the dynamics of antibody-mediated immune responses to SARS-CoV-2 infection is important for tracking and predicting Covid-19 disease progression and transmission, and for evaluating the effectiveness and durability of vaccination (11–14). We investigated the concurrent levels of IgG, IgM and IgA levels, and plasma anti-SARS-CoV-2 neutralizing ability against three viral variants, in subjects with diverse infection and BNT162b2 vaccination histories.

The primary findings of this observational study were that all three experimental groups developed primarily IgG-associated antibody responses against SARS-CoV-2 antigens with varying timing and extent after infection or BNT162b2 vaccination, and neutralization against B.1, Gamma, and Delta variants paralleled these responses. When comparing IgG levels, individuals who had recovered from Covid-19 and then received a single vaccination had the highest anti-RBD and anti-S1 antibody levels than unvaccinated individuals experiencing Covid-19 symptoms and Covid-naïve persons who received both doses of the standard vaccination schedule, through the timepoints we tracked. Indeed, anti-RBD and anti-S1 IgG levels measured 10 days after vaccination were respectively twice and 8-fold greater in the Covid-recovered/1 Vaccine Dose group than antigen-specific IgG levels measured in Covid-19 patients 14 days after symptom onset. This supports prior observations that SARS-CoV-2 exposure naturally primes the immune system and enhances both the speed and extent of IgG elevation after administering a single vaccine dose (24, 25). In a nested case-control study of 51 health-care workers in London who all received a single BNT162b2 vaccination, 24 of whom had prior SARS-CoV-2 exposure, total anti-S1 antibody levels measured a median 22 days post-vaccination (using the Elecsys anti-SARS-CoV-2 spike ECLIA, Roche Diagnostics) were 140-fold higher in the previously infected group (25). Another group compared anti-S1 IgG seroconversion in 67 SARS-CoV-2-seronegative and 43 seropositive individuals (mean age 41-years-old in both groups) after vaccination with either BNT162b2 (n=88) or mRNA-1273 (n=22; Moderna) (24). In Covid-naïve subjects, anti-spike IgG levels (provided as median AUC units) increased from 1 at baseline to 1293 units at 21-27 days after first vaccination, and to 3316 units after the second vaccine dose. In contrast, participants with evidence of prior SARS-CoV-2 exposure displayed median anti-spike IgG levels of 90 AUC units at baseline, which rapidly and uniformly elevated to a maximum of 25,927 units at 13-16 days after vaccination, and remained at 22,507 after the second vaccine dose. Thus, pre-exposure to SARS-CoV-2 resulted in faster and more pronounced anti-spike IgG generation than occurred in Covid-naïve subjects, and second vaccine doses did not further increase anti-spike IgG levels in individuals with pre-existing immunity. Anti-spike IgG dynamic responses that were elicited by a single vaccine dose did not significantly differ between the two mRNA vaccines. In our study, maximum anti-S1 IgG levels were identified in Covid-19-recovered individuals 8‑10 days after a single BNT162b2 vaccination; however, in Covid-naïve subjects, significantly increased anti-S1 IgG was only detected 14 days after the first vaccine dose and was maximally increased 25-28 days after the first dose (4‑7 days after the second vaccine dose).

An Italian group evaluated nAb production and T- and B-cell responses after BNT162b2 vaccination in 22 individuals, 11 with a history of prior SARS-CoV-2 infection (26). That study confirmed that a single dose of BNT162b2 enhanced both humoral and cellular immune responses against SARS-CoV-2, that were not additionally increased by a second vaccine dose in people who had prior exposure and a measure of natural immunity. A second vaccination was deemed mandatory in Covid-19-naïve individuals who even 3‑4 weeks after their second dose had antibody levels lower than Covid-recovered individuals who received a single dose. These observations were verified in an extended cohort of Covid-naïve (n=68) and Covid-recovered (n=29) subjects who were assessed for up to 50 days after vaccination, and helped guide the decision by public health officials in nations including Italy, France, and Germany to administer a single vaccine dose to some people who had previously been exposed to and recovered from SARS‑CoV-2, as a means to maximize the benefit of a limited vaccine supply. Similar findings regarding the effectiveness of a single SARS-CoV-2 vaccine dose in eliciting anti-spike IgG in previously exposed persons have since been reported by other researchers (35, 36), and immunological memory may last as long as 11 months (37). While anti-SARS-CoV-2 antibody production levels after infection correlate with Covid-19 symptom severity (27, 38), a study of healthcare workers reported no effect of Covid-19 severity on subsequent post-vaccination antibody responses (39). Taken together, the findings by us and others indicate a robust IgG response against SARS‑CoV‑2 antigens after a single BNT162b2 vaccination in previously exposed individuals (28), that matches or even exceeds that observed in Covid-naïve individuals who receive the complete two-vaccine series. However, the durability of antibody responses after BNT162b2 vaccination requires clarification. This is highlighted by the observation that anti‑RBD antibody levels decayed by more than 50% between 40 and 89 days after 2^nd^ dose administration of the 2-dose series, including in people who had previously recovered from mild Covid-19 (40).

Plasma levels of anti-spike IgA in persons with Covid-19 appear to parallel disease severity (12, 13, 41). The function of plasma IgA antibodies against SARS-CoV-2 is uncertain because they may not reach infection sites in the respiratory tract and IgA lacks IgG’s effector function in antibody-dependent cellular cytotoxicity and complement activation (42), although serum IgA levels in Covid-19 patients correlated with neutralizing capacity (13). In a prior study, serum IgA was transiently positive in subjects with mild Covid-19,and high levels were associated with severe respiratory distress syndrome (12). Some subjects with negative SARS‑CoV-2–specific serum IgA showed virus-specific neutralizing IgA in mucosal fluids, with levels inversely correlated with age. Secretory IgA can inhibit pathogen adherence to the mucosal lining (43). Secreted IgA also induces inflammatory chemokine (*e.g.*, IL-6, IL-8, MCP‑1, GM-CSF) production in lung fibroblasts, and may thereby influence the trajectory of SARS-CoV-2 pathogenesis (44). In the current study, we saw a trend of increasing anti-RBD IgA, but not anti-S1 IgA at Days 7 and 14 after symptom onset in unvaccinated subjects hospitalized with Covid-19. In the Covid-naïve/2 Vaccine Doses group anti-RBD IgA levels were significantly elevated by 11 days after the first vaccination and then increased steeply in the week after the second vaccination, becoming maximal at Day 28, after which levels slowly declined through the 42-day follow-up. Anti-S1 IgA was only significantly and transiently elevated between Days 28 and 32 after first vaccination (7‒11 days after 2^nd^ vaccine dose) in these subjects. In Covid-recovered/1 Vaccine Dose individuals, plasma anti‑RBD IgA levels appeared to increase by Day 6 post-vaccination and appeared to be maximal at Days 8‑10, with IgA levels approximately twice the maximum level seen in the Covid-naïve/2 Vaccine Dose group; however, our study was not sufficiently powered to assign statistical significance to this apparent IgA increase. No obvious increase in anti-S1 IgA was noted after single-dose vaccination in Covid-19-recovered subjects. A robust elevation in S1-reactive IgA was previously reported starting 14 days after BNT162b2 vaccination in Covid-naïve subjects and 7 days after vaccination in Covid-recovered individuals, with similar maximum levels maintained beyond 28 days (26). Differences between those findings and ours could be due to different patient populations and testing methodologies. The functional role of serum versus mucosal IgA responses following Covid‑19 infection and SARS-CoV-2 vaccination requires additional clarification.

Serum levels of SARS-CoV-2-reactive IgM rise heterogeneously among patients with Covid-19, with median seroconversion times reportedly varying between 5 and 13 days post‑symptom onset and persisting for at least 3 weeks (45, 46). Following BNT162b2 vaccination, anti-spike IgM in Covid-naïve individuals reportedly becomes significantly elevated by Day 14, dips again below detection by Day 21, and is again elevated by Day 28 (approximately 1 week following the 2^nd^ vaccine dose) (26). In Covid-recovered individuals, vaccination transiently increased anti-spike IgM around Day 21. In our study, large heterogeneity in IgM responses were observed. Only low levels of anti-S1 IgM were detected in any of our three subject groups. Anti-RBD IgM numerically increased by 3-fold in hospitalized Covid-19 subjects by Day 14, but this was not significant. In the Covid-naïve/2 Vaccine Doses group, anti‑RBD IgM appeared to transiently increase to maximal levels at Day 14, but this change was not mathematically significant. In Covid‑recovered/1 Vaccine Dose subjects, anti-RBD IgM levels remained at or below the assay limits of quantification through the 10-day follow-up. Thus, we were unable to discern a clear viral-specific IgM response to SARS-CoV-2 infection or to BNT162b2 vaccination in our subjects. Expanded antibody isotyping studies may elucidate IgM involvement in Covid-19 pathogenesis and responses to vaccination.

*In vitro* neutralization assays of SARS-CoV-2 infectivity by patient plasma provide important evidence on the effectiveness of a humoral response against specific viral variants (47). Valid concerns have arisen that certain SARS-CoV-2 strains such as the Gamma and Delta variants may evolve the ability to at least partially evade natural and vaccine‑initiated humoral immune responses (29, 31). A study of 180 Finnish healthcare workers indicated that sera after 2-dose BNT162b2 vaccination effectively neutralized the D614G variant FIN-25 (identified in Spring 2020) representing the B.1 lineage and the Alpha variant (B.1.1.7; UK origin) but was only 20% as effective against the Beta variant (B.1.351, South Africa origin) (30). However, the IC_50%_ neutralization titer against B.1.351 was still >1:20, and more than 92% of participants displayed measurable nAb titers against the B.1.351 variant, together indicating maintenance of significant protection against this variant. In a case-control study of nearly 16,000 British citizens who received two doses of BNT162b2, vaccination provided 88.0% against the Delta variant (B.1.617.2, India origin) (48). In our study, viral-specific IgG levels, especially those directed against the RBD, seemed well-correlated with plasma neutralizing ability against SARS‑CoV-2 variants. By far the highest neutralizing titers were observed in previously infected subjects who received a single vaccination after recovering from Covid-19; these individuals developed significantly higher neutralization titers against B.1 and Gamma strains compared to Covid-19-naïve individuals who received two vaccine doses. Although neutralizing activity against the Delta variant was only 26% of that observed in the B.1 wild type-like parental strain in the Covid-19-recovered/1 Vaccine group, this titer was higher than the maximum neutralizing titers observed against all three SARS-CoV-2 variants in both the Covid‑19‑recovered/Unvaccinated group and the Covid-19-naïve/2 Vaccine subjects. Natural immunity in unvaccinated Covid-19 patients and that provided by the 2‑dose BNT162b2 series appeared to provide similar neutralizing ability against the B.1 strain, and Gamma (P.1, Brazil origin) and Delta variants. The lowest study-wide IC_50_ neutralizing titer measured against the Delta variant was in Covid-19-recovered/Unvaccinated individuals relying on natural immunity alone, but still averaged 1:75, suggesting significant protection. Additionally, it appeared that anti-RBD IgG and possibly anti-S1 IgG were still increasing in Covid‑19‑recovered/Unvaccinated individuals at our latest observation point following symptom onset, so final nAb levels may have ultimately been higher. Antibody affinity maturation has been suggested as a mechanism by which plasma neutralizing titers have been found to increase in some individuals even as neutralizing anti-RBD IgG levels decline over time (4). Such immune enhancement may become increasingly important as data emerge indicating limited durability of the antibody response to SARS-CoV-2 mRNA vaccination (40, 49).

This study solidifies and extends our appreciation of the protective benefits of BNT162b2 vaccination and expands our understanding of the underlying antibody-mediated immune mechanisms. Study strengths include the use of subject populations from an early European Covid-19 epicenter and the investigation of multiple SARS-CoV-2 variants of concern. Primary study limitations include the focus on early time periods after infection/vaccination that cannot provide information on the durability of protection, relatively small group n‑values that preclude more rigorous statistical evaluation, and the restriction of studying humoral but not T‑cell‑mediated immunity. Nonetheless, our current findings support the usefulness of mRNA vaccination against Covid-19 for adults regardless of their SARS-CoV-2 exposure history, and particularly highlight the remarkable level of protection provided by a single vaccine dose in people who have experienced and recovered from Covid-19. This protection extends across distinct SARS-CoV-2 variants of concern including the increasingly prevalent Delta strain.

## Data Availability Statement

The datasets presented in this study can be found in online repositories. The names of the repository/repositories and accession number(s) can be found below: https://www.gisaid.org/, accession IDs: EPI_ISL_1085167, EPI_ISL_2472918, and EPI_ISL_2840619 for Alpha, Gamma, and Delta variants, respectively.

## Ethics Statement

The studies involving human participants were reviewed and approved by Luigi Sacco Hospital Institutional Review Board, University of Milan. The patients/participants provided their written informed consent to participate in this study.

## Author Contributions

Conceptualization: MT and WJ. Subject Enrollment and Clinical Assessment: AR, MS, AC, and MC. Sample Collection and Processing: AG and CV. Laboratory Methodology: MT, AB, CV, and WJ. Funding Acquisition: WJ. Data Analysis: MT and WJ. Study Management: MT, MG, and SA. Writing – original draft: MT and WJ. Writing – review & editing: All authors. All authors contributed to the article and approved the submitted version.

## Funding

This study was funded by Luminex Corp., Austin, TX. The funder was not involved in the study design, collection, analysis, interpretation of data, the writing of this article or the decision to submit it for publication.

## Conflict of Interest

WD was an employee of Luminex Corporation during the study period.

The remaining authors declare that the research was conducted in the absence of any commercial or financial relationships that could be construed as a potential conflict of interest.

## Publisher’s Note

All claims expressed in this article are solely those of the authors and do not necessarily represent those of their affiliated organizations, or those of the publisher, the editors and the reviewers. Any product that may be evaluated in this article, or claim that may be made by its manufacturer, is not guaranteed or endorsed by the publisher.

## References

[1] Cucinotta D Vanelli M. WHO Declares COVID-19 a Pandemic. Acta BioMed (2020) 91(1):157‒60. doi: 10.23750/abm.v91i1.9397 32191675 PMC7569573

[2] Dong E Du H Gardner L. An Interactive Web-Based Dashboard to Track COVID-19 in Real Time. Lancet Infect Dis (2020) 20:533‒4. doi: 10.1016/S1473-3099(20)30120-1 32087114 PMC7159018

[3] Johns Hopkins University, Center for Science and Systems Engineering. Coronavirus Resource Center (2021). Available at: https://coronavirus.jhu.edu/map.html (Accessed October 11, 2021).

[4] Piccoli L Park YJ Tortorici MA Czudnochowski N Walls AC Beltramello M. Mapping Neutralizing and Immunodominant Sites on the SARS-CoV-2 Spike Receptor-Binding Domain by Structure-Guided High-Resolution Serology. Cell (2020) 183(4):1024‒42.e21. doi: 10.1016/j.cell.2020.09.037 32991844 PMC7494283

[5] Walls AC Park YJ Tortorici MA Wall A McGuire AT Veesler D. Structure, Function, and Antigenicity of the SARS-CoV-2 Spike Glycoprotein. Cell (2020) 181(2):281‒92.e6. doi: 10.1016/j.cell.2020.02.058 32155444 PMC7102599

[6] Shang J Ye G Shi K Wan Y Luo C Aihara H. Structural Basis of Receptor Recognition by SARS-CoV-2. Nature (2020) 581:221–4. doi: 10.1038/s41586-020-2179-y PMC7328981 32225175

[7] Hoffmann M Kleine-Weber H Schroeder S Krüger N Herrler T Erichsen S. SARS-CoV-2 Cell Entry Depends on ACE2 and TMPRSS2 and is Blocked by a Clinically Proven Protease Inhibitor. Cell (2020) 181(2):271‒80.e8. doi: 10.1016/j.cell.2020.02.052 32142651 PMC7102627

[8] Letko M Marzi A Munster V. Functional Assessment of Cell Entry and Receptor Usage for SARS-CoV-2 and Other Lineage B Betacoronaviruses. Nat Microbiol (2020) 5(4):562‒9. doi: 10.1038/s41564-020-0688-y 32094589 PMC7095430

[9] Lan J Ge J Yu J Shan S Zhou H Fan S. Structure of the SARS-CoV-2 Spike Receptor-Binding Domain Bound to the ACE2 Receptor. Nature (2020) 581(7807):215‒20. doi: 10.1038/s41586-020-2180-5 32225176

[10] Yan R Zhang Y Li Y Xia L Guo Y Zhou Q. Structural Basis for the Recognition of SARS-CoV-2 by Full-Length Human ACE2. Science (2020) 367(6485):1444‒8. doi: 10.1126/science.abb2762 32132184 PMC7164635

[11] Fraser DD Cepinskas G Slessarev M Martin CM Daley M Patel MA. Critically Ill COVID-19 Patients Exhibit Anti-SARS-CoV-2 Serological Responses. Pathophysiology (2021) 28(2):212‒23. doi: 10.3390/pathophysiology28020014 PMC8830473 35366258

[12] Cervia C Nilsson J Zurbuchen Y Valaperti A Schreiner J Wolfensberger A. Systemic and Mucosal Antibody Responses Specific to SARS-CoV-2 During Mild Versus Severe COVID-19. J Allergy Clin Immunol (2021) 147(2):545‒57.e9. doi: 10.1016/j.jaci.2020.10.040 33221383 PMC7677074

[13] Patil HP Rane PS Shrivastava S Palkar S Lalwani S Mishra AC. Antibody (IgA, IgG, and IgG Subtype) Responses to SARS-CoV-2 in Severe and Nonsevere COVID-19 Patients. Viral Immunol (2021) 34(3):201‒9. doi: 10.1089/vim.2020.0321 33656935

[14] Legros V Denolly S Vogrig M Boson B Siret E Rigaill J. A Longitudinal Study of SARS-CoV-2-Infected Patients Reveals a High Correlation Between Neutralizing Antibodies and COVID-19 Severity. Cell Mol Immunol (2021) 18(2):318‒27. doi: 10.1038/s41423-020-00588-2 33408342 PMC7786875

[15] Pradenas E Trinité B Urrea V Marfil S Ávila-Nieto C Rodríguez de la Concepción ML. Stable Neutralizing Antibody Levels 6 Months After Mild and Severe COVID-19 Episodes. Med (NY) (2021) 2(3):313‒20.e4. doi: 10.1016/j.medj.2021.01.005 PMC7847406 33554155

[16] Lucas C Klein J Sundaram ME Liu F Wong P Silva J. Delayed Production of Neutralizing Antibodies Correlates With Fatal COVID-19. Nat Med (2021) 27(7):1178‒86. doi: 10.1038/s41591-021-01355-0 33953384 PMC8785364

[17] Pfizer Manufacturing Belgium NV. Pfizer-BioNTech COVID-19 Vaccine: Fact Sheet for Healthcare Providers Administering Vaccine (Vaccination Providers) (2021). Available at: https://labeling.pfizer.com/ShowLabeling.aspx?id=14471 (Accessed October 10, 2021).

[18] Walsh EE Frenck RW Jr Falsey AR Kitchin N Absalon J Gurtman A. Safety and Immunogenicity of Two RNA-Based Covid-19 Vaccine Candidates. N Engl J Med (2020) 383(25):2439‒50. doi: 10.1056/NEJMoa2027906 33053279 PMC7583697

[19] Sahin U Muik A Vogler I Derhovanessian E Kranz LM Vormehr M. BNT162b2 Vaccine Induces Neutralizing Antibodies and Poly-Specific T Cells in Humans. Nature (2021) 595(7868):572‒7. doi: 10.1038/s41586-021-03653-6 34044428

[20] Polack FP Thomas SJ Kitchin N Absalon J Gurtman A Lockhart S. Safety and Efficacy of the BNT162b2 mRNA Covid-19 Vaccine. N Engl J Med (2020) 383(27):2603‒615. doi: 10.1056/NEJMoa2034577 33301246 PMC7745181

[21] United States Food and Drug Administration. “FDA Takes Key Action in Fight Against COVID-19 by Issuing Emergency Use Authorization for First COVID-19 Vaccine.” News Release of the Food and Drug Administration, Silver Spring, Md (2020). Available at: https://www.fda.gov/news-events/press-announcements/fda-takes-key-action-fight-against-covid-19-issuing-emergency-use-authorization-first-covid-19 (Accessed October 10, 2021).

[22] Frenck RW Jr Klein NP Kitchin N Gurtman A Absalon J Lockhart S. Safety, Immunogenicity, and Efficacy of the BNT162b2 Covid-19 Vaccine in Adolescents. N Engl J Med (2021) 385(3):239‒50. doi: 10.1056/NEJMoa2107456 34043894 PMC8174030

[23] United States Food and Drug Administration. “Coronavirus (COVID-19) Update: FDA Authorizes Pfizer-BioNTech COVID-19 Vaccine for Emergency Use in Adolescents in Another Important Action in Fight Against Pandemic.”, in: News Release of the Food and Drug Administration, Silver Spring, Md (2021). Available at: https://www.fda.gov/news-events/press-announcements/coronavirus-covid-19-update-fda-authorizes-pfizer-biontech-covid-19-vaccine-emergency-use (Accessed October 10, 2021). May 10, 2021.

[24] Krammer F Srivastava K Alshammary H Amoako AA Awawda MH Beach KF. Antibody Responses in Seropositive Persons After a Single Dose of SARS-CoV-2 mRNA Vaccine. N Engl J Med (2021) 384(14):1372‒74. doi: 10.1056/NEJMc2101667 33691060 PMC8008743

[25] Manisty C Otter AD Treibel TA McKnight Á Altmann DM Brooks T. Antibody Response to First BNT162b2 Dose in Previously SARS-CoV-2-Infected Individuals. Lancet (2021) 397(10279):1057‒8. doi: 10.1016/S0140-6736(21)00501-8 33640038 PMC7972310

[26] Mazzoni A Di Lauria N Maggi L Salvati L Vanni A Capone M. First-Dose mRNA Vaccination is Sufficient to Reactivate Immunological Memory to SARS-CoV-2 in Subjects Who Have Recovered From COVID-19. J Clin Invest (2021) 131(12):e149150. doi: 10.1172/JCI149150 PMC8203460 33939647

[27] Ibarrondo FJ Fulcher JA Goodman-Meza D Elliott J Hofmann C Hausner MA. Rapid Decay of Anti-SARS-CoV-2 Antibodies in Persons With Mild Covid-19. N Engl J Med (2020) 383(11):1085-7. doi: 10.1056/NEJMc2025179 32706954 PMC7397184

[28] Frieman M Harris AD Herati RS Krammer F Mantovani A Rescigno M. SARS-CoV-2 Vaccines for All But a Single Dose for COVID-19 Survivors. EBioMedicine (2021) 68:103401. doi: 10.1016/j.ebiom.2021.103401 34051441 PMC8149267

[29] Nadesalingam A Cantoni D Wells DA Aguinam ET Ferrari M Smith P. Paucity and Discordance of Neutralising Antibody Responses to SARS-CoV-2 VOCs in Vaccinated Immunodeficient Patients and Health-Care Workers in the UK. Lancet Microbe (2021) 2(9):e416–8. doi: 10.1016/S2666-5247(21)00157-9 PMC8238451 34223399

[30] Jalkanen P Kolehmainen P Häkkinen HK Huttunen M Tähtinen PA Lundberg R. COVID-19 mRNA Vaccine Induced Antibody Responses Against Three SARS-CoV-2 Variants. Nat Commun (2021) 12(1):3991. doi: 10.1038/s41467-021-24285-4 34183681 PMC8239026

[31] Planas D Veyer D Baidaliuk A Staropoli I Guivel-Benhassine F Rajah MM. Reduced Sensitivity of SARS-CoV-2 Variant Delta to Antibody Neutralization. Nature (2021) 596(7871):276‒80. doi: 10.1038/s41586-021-03777-9 34237773

[32] Zehender G Lai A Bergna A Meroni L Riva A Balotta C. Genomic Characterization and Phylogenetic Analysis of SARS-COV-2 in Italy. J Med Virol (2020) 92(9):1637‒40. doi: 10.1002/jmv.25794 32222993 PMC7228393

[33] Mouffak S Shubbar Q Saleh E El-Awady R. Recent Advances in Management of COVID-19: A Review. BioMed Pharmacother (2021) 143:112107. doi: 10.1016/j.biopha.2021.112107 34488083 PMC8390390

[34] Vashi AP Coiado OC. The Future of COVID-19: A Vaccine Review. J Infect Public Health (2021) 14(10):1461–5. doi: 10.1016/j.jiph.2021.08.011 PMC8363422 34454862

[35] Efrati S Catalogna M Hamad RA Hadanny A Bar-Chaim A Benveniste-Levkovitz P. Safety and Humoral Responses to BNT162b2 mRNA Vaccination of SARS-CoV-2 Previously Infected and Naive Populations. Sci Rep (2021) 11(1):16543. doi: 10.1038/s41598-021-96129-6 34400714 PMC8367980

[36] Ebinger JE Fert-Bober J Printsev I Wu M Sun N Prostko JC. Antibody Responses to the BNT162b2 mRNA Vaccine in Individuals Previously Infected With SARS-CoV-2. Nat Med (2021) 27(6):981‒4. doi: 10.1038/s41591-021-01325-6 33795870 PMC8205849

[37] Ferrari D Di Resta C Tomaiuolo R Sabetta E Pontillo M Motta A. Long-Term Antibody Persistence and Exceptional Vaccination Response on Previously SARS-CoV-2 Infected Subjects. Vaccine (2021) 39(31):4256‒60. doi: 10.1016/j.vaccine.2021.06.020 34147292 PMC8196312

[38] Chen X Pan Z Yue S Yu F Zhang J Yang Y. Disease Severity Dictates SARS-CoV-2-Specific Neutralizing Antibody Responses in COVID-19. Signal Transduct Target Ther (2020) 5(1):180. doi: 10.1038/s41392-020-00301-9 32879307 PMC7464057

[39] Vicenti I Gatti F Scaggiante R Boccuto A Zago D Basso M. Single-Dose BNT162b2 mRNA COVID-19 Vaccine Significantly Boosts Neutralizing Antibody Response in Health Care Workers Recovering From Asymptomatic or Mild Natural SARS-CoV-2 Infection. Int J Infect Dis (2021) 108:176‒8. doi: 10.1016/j.ijid.2021.05.033 34022329 PMC8132552

[40] Erice A Varillas-Delgado D Caballero C. Decline of Antibody Titres Three Months After Two Doses of BNT162b2 in non-Immunocompromised Adults. Clin Microbiol Infect (2021) S1198-743X(21). doi: 10.1016/j.cmi.2021.08.023 PMC8426320 34508885

[41] Yu HQ Sun BQ Fang ZF Zhao JC Liu XY Li YM. Distinct Features of SARS-CoV-2-Specific IgA Response in COVID-19 Patients. Eur Respir J (2020) 56(2):2001526. doi: 10.1183/13993003.01526-2020 32398307 PMC7236821

[42] Valdez-Cruz NA García-Hernández E Espitia C Cobos-Marín L Altamirano C Bando-Campos CG. Integrative Overview of Antibodies Against SARS-CoV-2 and Their Possible Applications in COVID-19 Prophylaxis and Treatment. Microb Cell Fact (2021) 20(1):88. doi: 10.1186/s12934-021-01576-5 33888152 PMC8061467

[43] Corthésy B. Role of Secretory IgA in Infection and Maintenance of Homeostasis. Autoimmun Rev (2013) 12(6):661‒5. doi: 10.1016/j.autrev.2012.10.012 23201924

[44] Arakawa S Suzukawa M Watanabe K Kobayashi K Matsui H Nagai H. Secretory Immunoglobulin A Induces Human Lung Fibroblasts to Produce Inflammatory Cytokines and Undergo Activation. Clin Exp Immunol (2019) 195(3):287‒301. doi: 10.1111/cei.13253 30570135 PMC6378381

[45] Guo L Ren L Yang S Xiao M Chang D Yang F. Profiling Early Humoral Response to Diagnose Novel Coronavirus Disease (COVID-19). Clin Infect Dis (2020) 71(15):778‒85. doi: 10.1093/cid/ciaa31 32198501 PMC7184472

[46] Long QX Liu BZ Deng HJ Wu GC Deng K Chen YK. Antibody Responses to SARS-CoV-2 in Patients With COVID-19. Nat Med (2020) 26(6):845‒8. doi: 10.1038/s41591-020-0897-1 32350462

[47] Khoury DS Wheatley AK Ramuta MD Reynaldi A Cromer D Subbarao K. Measuring Immunity to SARS-CoV-2 Infection: Comparing Assays and Animal Models. Nat Rev Immunol (2020) 20(12):727‒38. doi: 10.1038/s41577-020-00471-1 33139888 PMC7605490

[48] Lopez Bernal J Andrews N Gower C Gallagher E Simmons R Thelwall S. Effectiveness of Covid-19 Vaccines Against the B.1.617.2 (Delta) Variant. N Engl J Med (2021) 385(7):585‒94. doi: 10.1056/NEJMoa2108891 34289274 PMC8314739

[49] Israel A Shenhar Y Green I Merzon E Golan-Cohen A Schäffer AA. Large-Scale Study of Antibody Titer Decay Following BNT162b2 mRNA Vaccine or SARS-CoV-2 Infection. medRxiv (2021). doi: 10.1101/2021.08.19.21262111 PMC8781423 35062724

